# Effect of the MPT Pore Inhibitor Alisporivir on the Development of Mitochondrial Dysfunction in the Heart Tissue of Diabetic Mice

**DOI:** 10.3390/biology10090839

**Published:** 2021-08-28

**Authors:** Natalia V. Belosludtseva, Vlada S. Starinets, Irina B. Mikheeva, Dmitriy A. Serov, Maxim E. Astashev, Maxim N. Belosludtsev, Mikhail V. Dubinin, Konstantin N. Belosludtsev

**Affiliations:** 1 Laboratory of Mitochondrial Transport, Institute of Theoretical and Experimental Biophysics, Russian Academy of Sciences, Institutskaya 3, 142290 Pushchino, Russia; nata.imagination@gmail.com (N.V.B.); vlastar@list.ru (V.S.S.); mikheirina@yandex.ru (I.B.M.); 2 Department of Biochemistry, Cell Biology and Microbiology, Mari State University, pl. Lenina 1, 424001 Yoshkar-Ola, Russia; bemanik89@gmail.com (M.N.B.); dubinin1989@gmail.com (M.V.D.); 3 Prokhorov General Physics Institute of the Russian Academy of Sciences, 38 Vavilove St., 119991 Moscow, Russia; dmitriy_serov_91@mail.ru (D.A.S.); astashev@yandex.ru (M.E.A.)

**Keywords:** alisporivir, mitochondria, diabetes mellitus, mitochondrial dysfunction, mitophagy, mitochondrial permeability transition pore

## Abstract

**Simple Summary:**

Diabetes mellitus as a systemic metabolic disease is one of the most serious threats to global health in this century. Diabetic cardiomyopathy is increasingly recognized as one of the most important complications of the disease, which is associated with impaired cell energy metabolism and damage to mitochondria in cardiomyocytes. Therefore, targeting mitochondrial dysfunction by pharmacological agents can be used as a therapeutic strategy in diabetic heart disease. The aim of the work was to study the effect of the mitochondria-targeted agent alisporivir on the development of mitochondrial dysfunction in the heart of mice with experimental diabetes mellitus. Alisporivir has been recently identified as a non-immunosuppressive analogue of cyclosporin A, a selective inhibitor of cyclophilin D and the mitochondrial permeability transition pore opening, with a potential in a wide range of therapeutic indications. Our results indicated that alisporivir alleviates diabetes-induced abnormalities in the ultrastructure and functions of mitochondria in cardiomyocytes and increases the rate of glucose utilization in diabetic mice. The data suggest that alisporivir acts as a mitochondria-targeted metabolic reprogramming agent and attenuates oxidative damage to the heart tissue of diabetic mice.

**Abstract:**

Diabetes mellitus is a systemic metabolic disorder associated with mitochondrial dysfunction, with the mitochondrial permeability transition (MPT) pore opening being considered as one of its possible mechanisms. The effect of alisporivir, a non-immunosuppressive cyclosporin derivative and a selective inhibitor of the MPT pore opening, on the ultrastructure and functions of the heart mitochondria of mice with diabetes mellitus induced by a high-fat diet combined with streptozotocin injections was studied. The treatment of diabetic animals with alisporivir (2.5 mg/kg ip for 20 days) increased the rate of glucose clearance during the glucose tolerance test. The blood glucose level and the indicator of heart rate in alisporivir-treated diabetic mice tended to restore. An electron microscopy analysis showed that alisporivir prevented mitochondrial swelling and ultrastructural alterations in cardiomyocytes of diabetic mice. Alisporivir canceled the diabetes-induced increases in the susceptibility of heart mitochondria to the MPT pore opening and the level of lipid peroxidation products, but it did not affect the decline in mitochondrial oxidative phosphorylation capacity. The mRNA expression levels of *Pink1* and *Parkin* in the heart tissue of alisporivir-treated diabetic mice were elevated, suggesting the stimulation of mitophagy. In parallel, alisporivir decreased the level of mtDNA in the heart tissue. These findings suggest that targeting the MPT pore opening by alisporivir alleviates the development of mitochondrial dysfunction in the diabetic heart. The cardioprotective effect of the drug in diabetes can be mediated by the induction of mitophagy and the inhibition of lipid peroxidation in the organelles.

## 1. Introduction

Diabetes mellitus (DM) as a metabolic disease is one of the most serious threats to human health throughout the world. It is based on hyperglycemia and insufficient insulin secretion or malfunction. These metabolic disturbances lead to damage to many organs and tissues of the organism. One of the most common complications of diabetes mellitus is cardiomyopathy [1,2,3]. Diabetic cardiomyopathy is defined as the presence of abnormal myocardial structure and function in patients with DM, with the exclusion of hypertension, coronary artery disease, and other pre-existing cardiovascular diseases [4]. Diabetes-induced changes in cardiomyocytes result in a wide range of structural and biochemical abnormalities eventually leading to systolic and diastolic dysfunction. One of these abnormalities is mitochondrial dysfunction in cardiomyocytes [4,5,6,7].

Diabetic cardiomyopathy is known to be closely associated with impaired mitochondrial function, dysregulation of mitophagy and mitochondrial biogenesis, pathological changes in the dynamics of the organelles, as well as oxidative stress [5,6]. It is important to note that mitochondria are one of the regulators of intracellular Ca^2+^ homeostasis [8]. Therefore, diabetes-induced mitochondrial dysfunction in the heart tissue also leads to impaired intercellular Ca^2+^ handling. Indeed, the development of diabetes mellitus was marked by a decrease in the rate of Ca^2+^ uptake by cardiac mitochondria due to the reduction of MCU, a channel subunit of the mitochondrial Ca^2+^ uniporter complex [9,10,11,12]. Along with this, there is the development of a pathology-associated mitochondrial permeability transition (MPT) pore opening in the inner membrane of organelles of cardiomyocytes [7,8].

The MPT pore is a calcium-activated multiprotein channel that permeates both the inner and outer mitochondrial membranes. The opening of the MPT pore makes the inner mitochondrial membrane permeable to compounds with molecular weights up to 1.5 kDa, causing ion imbalance, mitochondrial depolarization, and swelling of the organelles. Subsequently, these processes can result in a decline in the cell’s energy supply, mitochondrial and cellular destruction [8,13]. It should be noted that the structural composition of the MPT pore channel is still under discussion. Recent data suggest that ATP-synthase and adenylate translocator are the best molecular candidates for the MPT pore formation. The only protein whose reliability of participation in the pore formation is not questioned is its regulator cyclophilin D [8,14,15]. In this sense, genetic and pharmacological modification of this cornerstone protein is a tool in assessing the role of the MPT pore in the pathogenesis of diabetic cardiomyopathy [15,16,17].

As mentioned above, diabetic cardiomyopathy is accompanied by a decrease in mitochondrial resistance to the MPT pore induction [7,10,18,19]. Therefore, the correction of this pathology with the MPT pore inhibitors looks attractive. At the same time, the results of the MPT pore opening correction in diabetes mellitus are rather contradictory. Thus, on the one hand, it was shown that administration of MPT pore inhibitors (NIM811, MTP-131, cyclosporin A) or the mitochondrial calcium uniporter blocker minocycline at the onset of reperfusion reduced infarct sizes in both control and diabetic hearts [20,21]. These findings suggest that augmented susceptibility to injury in the diabetic heart is mediated by redox-dependent shifts in the MPT pore opening. On the other hand, it was demonstrated that inhibition of the MPT pore with cyclosporin A failed to restore cardioprotection in the prediabetic but normoglycemic heart of Zucker obese rats in vivo [22]. Moreover, cyclosporin A is known to suppress the immune system as well as inhibit calcineurin signaling [16], causing side effects in the development of diabetes mellitus. All this suggests the feasibility of studying the effect of various modulators of the MPT pore on diabetic cardiomyopathy.

Alisporivir (Ali) is a non-immunosuppressive analogue of cyclosporin A, inhibiting MPT pore assembly through interaction with cyclophilin D [23,24]. The literature describes that this effect of alisporivir underlies its protective action in ischemia/reperfusion of the heart [24], as well as in the development of muscular dystrophies [25,26]. Moreover, alisporivir is able to block the replication of a number of viruses, including SARS-CoV-2, by inhibiting the activity of cyclophilins in mammalian cells [27]. Diabetes mellitus is one of the concomitant diseases that causes severe complications in patients with COVID-19 [28]. All this allowed us to assume that this compound can have a protective effect on the cells and mitochondria of the cardiac muscles of diabetic animals. The aim of this work is to study the antidiabetic potential of alisporivir, a non-immunosuppressive inhibitor of the MPT pore, and its possible protective effect against mitochondrial injury in the heart tissue of C57BL/6NCrl mice with high-fat diet/streptozotocin-induced diabetes.

## 2. Materials and Methods

### 2.1. Animals and the Induction of Diabetes

**Figure 1:**
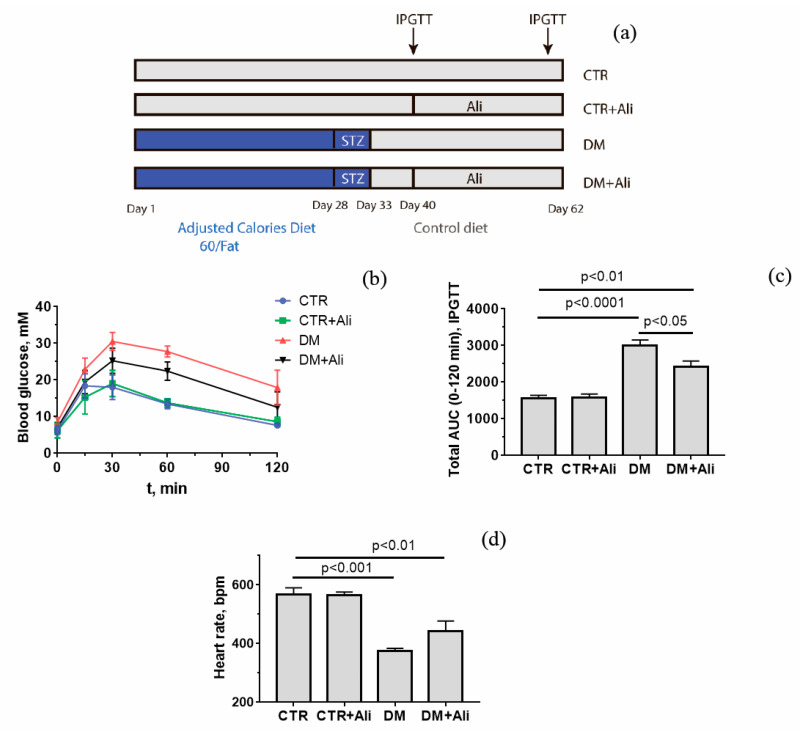
Scheme for induction of diabetes mellitus (**a**), intraperitoneal glucose tolerance test (**b**) in experimental group of animals. The total area under the curve (AUC) during IPGTT (**c**). Heart rate of animals in four experimental groups (**d**). The values are given as mean ± SEM (*n* = 5).

Male mice of the C57BL/6NCrl line weighing 23–26 g were used in this work. Mice were randomly divided into four groups: (1) non-treated control (CTR) (*n* = 9); (2) control + alisporivir (CTR + Ali) (*n* = 9); (3) mice with diabetes mellitus (DM) (*n* = 9); and (4) mice with DM treated with alisporivir (DM + Ali) (*n* = 9). DM was induced in animal groups 3 and 4 by high-fat diet feeding (Adjusted Calories Diet 60/Fat, Envigo, Indianapolis, IN, USA) for 28 days. Thereafter, mice received intraperitoneal (ip) injections of streptozotocin (STZ) (30 mg/kg) for five consecutive days during high-fat diet feeding [29]. The scheme of the experiments is shown in Figure 1A. The control groups 1 and 2 were provided with a low-fat control diet (Envigo, Indianapolis, IN, USA). Alisporivir (2.5 mg/kg/day, ip) was administered to animals of the second and fourth groups for 20 days starting from the 40th day. Alisporivir was dissolved in a mixture of DMSO, ethanol, and sterile saline (12.5:25:62.5 *v*/*v*%). The control animals received solvent alone. Successful development of diabetes mellitus was assessed by the intraperitoneal glucose tolerance test (IPGTT). Before testing, mice were fasted for 16 h (IPGTT) with free access to water. The blood glucose level was recorded using a One Touch Select Plus glucometer (LifeScan, Zug, Switzerland). The triglyceride level was estimated using a Multicare-in biochemistry analyzer (Biochemical Systems International Srl, Arezzo, Italy).

### 2.2. Electron Microscopy

Tissue samples of the basal inferior segment of the left ventricle of two hearts of each experimental group of animals were examined by electron microscopy analysis. Fixation of tissue samples was carried out in accordance with the conventional technique as described [29,30]. The preparations were photographed using a JEM-100B electron microscope (JEOL, Tokio, Japan) and analyzed using an Epson V700 scanner.

### 2.3. RNA Extraction and Quantitative Real-Time PCR

Total RNA was extracted from 50 mg of deep-frozen mouse cardiac tissue (the left ventricle) using an ExtractRNA kit (Evrogen, Moscow, Russia). The real-time PCR was performed on a DTLite5 amplifier (DNA-Technology LLC, Moscow, Russia) [29]. A Primer-BLAST tool was used to select and analyze gene-specific primers shown in Table 1 [31]. The expression of each studied gene was normalized to the level of *Rplp2* mRNA, and the quantitative assessment of the data was carried out using the comparative C_T_ method [32].

### 2.4. Mitochondrial DNA Estimation

Total DNA (nuclear and mtDNA) was isolated from 10 mg of the left ventricle myocardium of mice using a DNA-Extran 2 kit (Sintol, Moscow, Russia) [29]. The mtDNA content in one nanogram of the total DNA was evaluated by PCR [33] and quantified as mtDNA/nuclear DNA ratio. The *ND4* gene of the mitochondrial genome and the *Gapdh* gene of the nuclear one were used for this assay (*Nd4* DNA/*Gapdh* DNA). Gene-specific primers are shown in Table 1. A DTLite5 amplifier was used for real-time PCR.

### 2.5. Mitochondria Isolation and Determination of Functional Parameters

Mitochondria were isolated from heart tissue by differential centrifugation [34]. Final suspensions contained 20–30 mg of mitochondrial protein/mL, as determined by the Bradford method. The rate of O_2_ consumption by mitochondrial samples was estimated using Oxygraph-2k (Oroboros Instruments, Innsbruck, Austria) [35]. The reaction buffer contained 120 mM KCl, 5 mM NaH_2_PO_4_, 2.5 mM potassium malate, 2.5 mM potassium glutamate, and 10 mM HEPES–KOH (pH 7.4). Ca^2+^ transport by mitochondria was estimated with an arsenazo III indicator at 675–685 nm using a plate reader Tecan Spark 10M (Tecan Group Ltd, Männedorf, Switzerland) [30]. The reaction buffer contained 210 mM mannitol, 70 mM sucrose, 1 mM KH_2_PO_4_, 2.5 mM malate, 2.5 mM glutamate, 10 μM EGTA, 50 μM arsenazo III, and 10 mM HEPES-KOH (pH 7.4.). Intensity of lipid peroxidation in mitochondrial membranes was assessed by quantification of thiobarbituric acid-reactive substances (TBARS) represented by malondialdehyde and some other minor aldehyde species [29].

### 2.6. ECG

For electrocardiograms examinations, the animals were immobilized using combined anesthesia [36]. A mouse was held at rest until it lost a righting reflex and then was placed into an experimental chamber. EGCs were monitored in the II standard lead during 10 min with the SparkFun Single Lead Heart Rate Monitor (SparkFun Electronics, Niwot, CO, USA). Recordings were started only after a negative tail-pinching reflex test to ensure the depth of surgical anesthesia [37,38].

### 2.7. Statistical Analysis

The data were presented as mean ± SEM (*n* = 4–9). Differences between the groups were calculated using the GraphPad Prism 7.0 software and one-way analysis of variance (ANOVA) followed by the Tukey multiple comparison post hoc test.

### 2.8. Materials

Alisporivir was purchased from MedChemExpress (HY-12559, MedChemExpress, Monmouth Junction, NJ, USA). Standardized animal feeds (Adjusted Calories Diet 60/Fat and low-fat control diet) were purchased from Envigo (Envigo, Indianapolis, IN, USA). RNA extraction and quantitative real-time PCR reagents were from Evrogen (Evrogen, Moscow, Russia). DNA extraction reagents were from Sintol (Sintol, Moscow, Russia). All other reagents were purchased from Invitrogen or Sigma-Aldrich (Merck, St. Louis, MO, USA).

## 3. Results

### 3.1. Somatic and Biochemical Characteristics of Experimental Mice

Table 2 and Figure 1 show data on some of the somatic and biochemical parameters of the C57BL/6NCrl mice of four experimental groups. Diabetic animals have significant increases in blood glucose (fed state), malondialdehyde, and triglycerides. Administration of alisporivir to diabetic animals has no effect on these parameters, although there was a tendency for their recovery to the level of control animals (reduction of glucose, malondialdehyde, and triglycerides levels in blood by 1.21, 1.15, and 1.16 times, respectively) (Table 2). At the same time, it should be noted that, according to the glucose tolerance test, alisporivir increased the rate of glucose utilization in diabetic animals (Figure 1b,c).

The development of diabetes mellitus is known to be accompanied by a change in the cardiac phenotype. As shown in our work, the induction of diabetes causes a decrease in the heart weight relative to the weight of this organ in control animals (Table 2). This change was accompanied by bradycardia—a decrease in heart rate. Alisporivir treatment of diabetic animals caused a 1.18-fold increase in heart rate relative to the DM group, but this change was not significant (Figure 1d).

### 3.2. Effects of Alisporivir on Diabetes-Induced Changes in the Functioning of Heart Mitochondria

**Figure 2:**
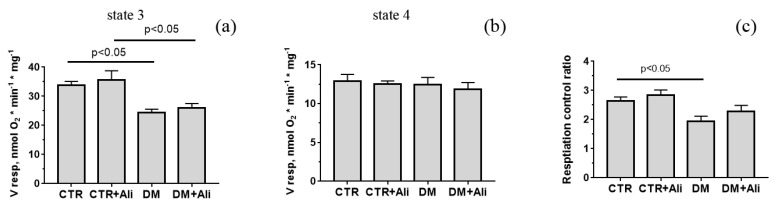
Parameters of respiration and oxidative phosphorylation of mouse heart mitochondria of the experimental groups. (**a**) The rate of respiration of mitochondria in state 3. (**b**) The rate of respiration of mitochondria in state 4. (**c**) Respiration control ratio. Medium composition: 130 mM KCl, 5 mM NaH_2_PO_4_, 10 µM EGTA, and 10 mM HEPES–KOH, pH 7.4. Respiration of mitochondria was fueled by 2.5 mM glutamate and 2.5 mM malate. Mitochondrial respiration in state 3 was initiated by 200 µM ADP. The results are presented as means ± SEM (*n* = 4).

Alisporivir is a specific inhibitor of the mitochondrial pore [23]. This property has been shown to determine the cardioprotective effect of alisporivir in ischemic heart damage [24]. In this work, we evaluated the effect of alisporivir on the functioning of cardiac mitochondria in diabetic cardiomyopathy. Figure 2 shows the data on the parameters of respiration and oxidative phosphorylation of cardiac mitochondria in mice from four experimental groups. One can see that the development of diabetes mellitus leads to a significant decrease (1.4-fold) in the rate of respiration in state 3 (ADP-stimulated), which, in turn, induces a decrease in the parameter of respiratory control compared to the CTR group. Alisporivir did not significantly change either the respiration rate of the heart mitochondria in diabetic mice or the parameter of respiratory control.

**Figure 3:**
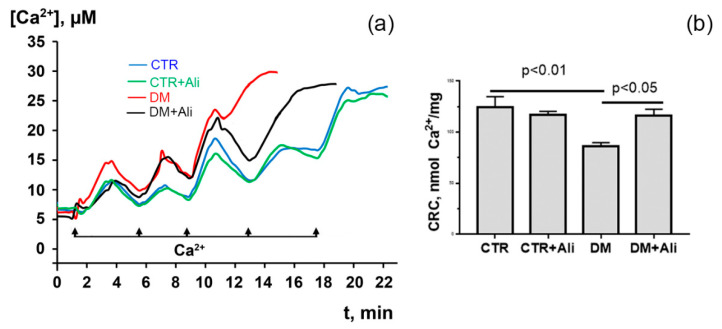
Changes in the external [Ca^2+^] upon successive addition of small Ca^2+^ doses (10 μM) to the suspension of heart mitochondria of experimental animals (**a**). Ca^2+^ retention capacity of heart mitochondria of experimental animals (**b**). The values are given as means ± SEM (*n* = 5).

At the same time, alisporivir, as a specific MPT pore blocker, significantly increased the resistance of diabetic mitochondria to its opening. Figure 3 presents data on the Ca^2+^ retention capacity of the heart mitochondria of mice in four experimental groups. It can be seen that the cardiac mitochondria of diabetic mice have a higher susceptibility to the pore induction (the calcium retention capacity parameter is reduced by 1.45 times) than the mitochondria of mice in the control group. Alisporivir administration to diabetic animals resulted in a significant increase in Ca^2+^ retention capacity.

**Figure 4:**
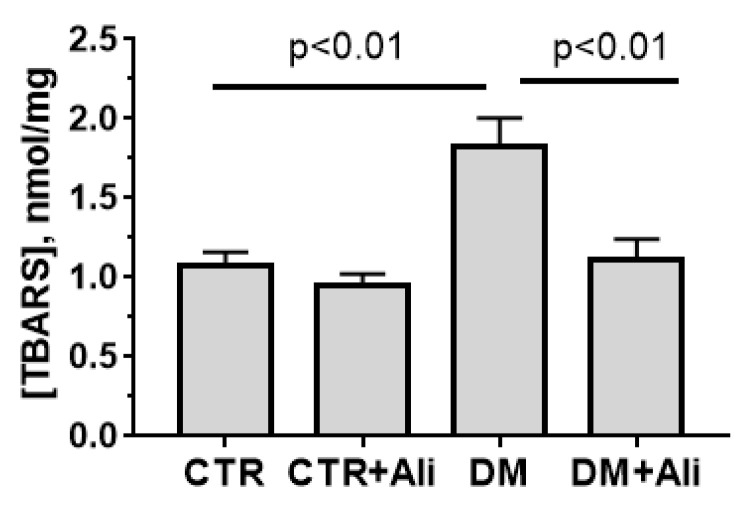
Alisporivir significantly suppresses the DM-induced lipid peroxidation in mouse heart mitochondria. Lipid peroxidation was assessed by the level of TBARS in the heart mitochondria of experimental groups of animals. Values are given as means ± SEM (*n* = 5).

Alisporivir treatment of diabetic animals also led to a decrease in the production of TBA-reactive substances (mainly, malondialdehyde) by mitochondria to the values of control animals (Figure 4). This indicates that alisporivir reduces the intensity of lipid peroxidation in the cardiac mitochondria of diabetic animals.

### 3.3. The Effect of Alisporivir on Ultrastructural Changes in Heart Mitochondria of Mice with Experimental DM and on the Expression of Protein Genes Responsible for Mitochondrial Biogenesis and Mitophagy

**Figure 5:**
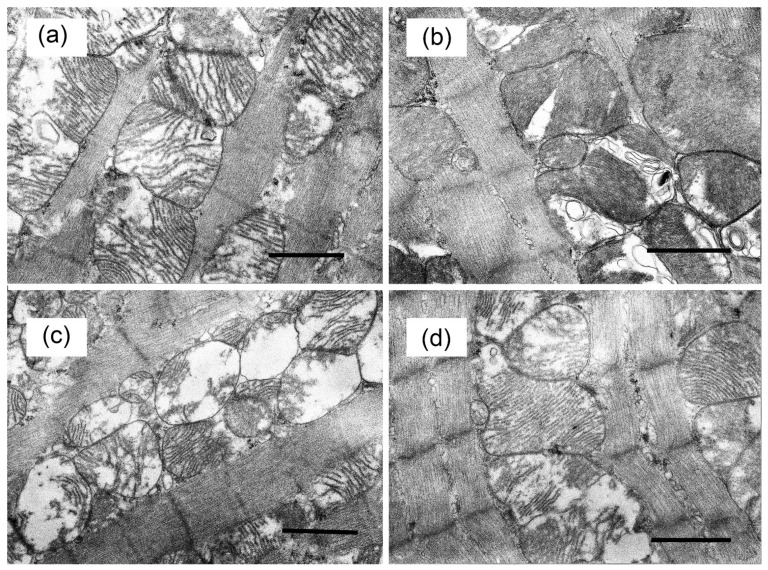
Typical electron micrographs of mouse heart mitochondria in the experimental groups: CTR (**a**), CTR + Ali (**b**), DM (**c**), and DM + Ali (**d**). The bar is equal to 1 μm.

Figure 5 shows representative photomicrographs of cardiac muscle cardiomyocytes of mice from the experimental groups. Electron microscopic examination of cardiomyocytes of the cardiac muscle in the CTR and CTR + Ali groups revealed an ordered arrangement of myofibrils with a correct transverse striation and a relatively dense packing of myofilaments. Mitochondria in the interfibrillar zone were arranged in a chain of 2–3 rows, forming close contacts with each other and were characterized by the regular organization of mitochondrial cristae. Specific destructive changes were found in the DM group. Most of the cardiomyocytes found dissociated mitochondria that did not have typical contacts. Some of the mitochondria showed complete destruction of the cristae, “watering” of the matrix, and their remains looked like large vacuoles. Expanded profiles of sarcoplasmic reticulum and glycogen accumulation could often be recorded. We also observed displacement of myofibrils in the contractile apparatus of cardiomyocytes, tortuosity of Z-disks and their mismatch in adjacent myofibrils. The DM + Ali group showed a slight dissociation in the arrangement of the muscle fiber bundles.

**Figure 6:**
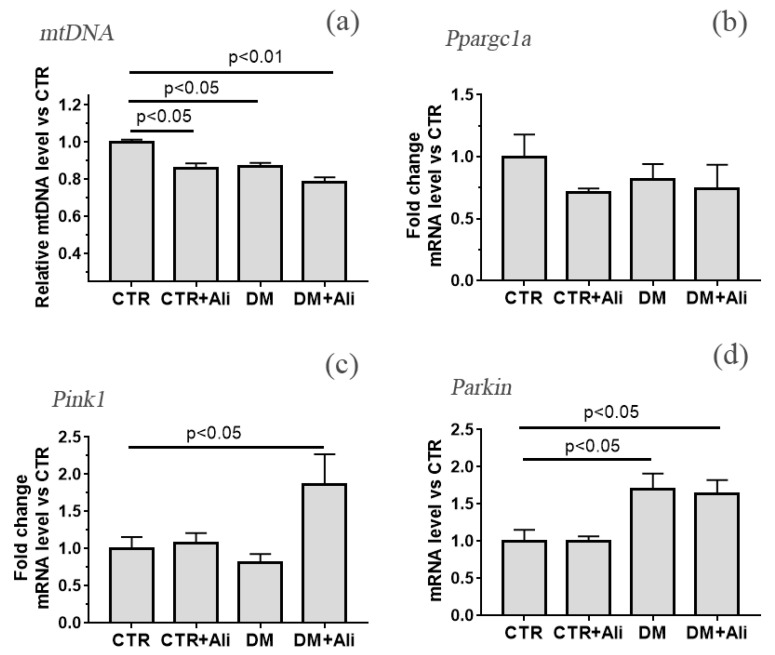
The relative mtDNA levels (**a**) and the mRNA levels of *Ppargc1a* (**b**), *Pink1* (**c**), *Parkin* (**d**) in the heart of experimental animals. The values are given as means ± SEM (*n* = 6).

We also determined the level of mitochondrial DNA in the left ventricular tissue, indirectly reflecting the number of mitochondria (Figure 6). One can see that the development of diabetes mellitus (DM group) is accompanied by a decrease in the amount of mitochondrial DNA (by about 15%) relative to the CTR group. The administration of alisporivir to diabetic animals led to an additional decrease in the level of mtDNA (~by 22% compared with the CTR group). One should note that alisporivir also reduced mtDNA level in healthy animals (CTR + Ali group).

The data obtained indicate that both the development of diabetes mellitus and the administration of alisporivir may lead to a decrease in the number of mitochondria in cardiomyocytes. This may be based on a change in the processes of mitochondrial biogenesis and mitophagy. Figure 6b–d shows the data on the relative expression level of the *Ppargc1a, Pink1*, and *Parkin* genes encoding the proteins responsible for the processes of mitochondrial biogenesis (PGC1α) and mitophagy (Pink1 and Parkin). We noted a downward trend in the *Ppargc1a* level in the CTR + Ali, DM, and DM + Ali groups (by 28, 20, and 27%, respectively) relative to the CTR group. In the DM group, there is a significant increase in the expression of the *Parkin* gene, but at the same time, a trend towards a decrease in the expression of the *Pink1* gene. The DM + Ali group showed a significant increase in the expression of the *Pink1* and *Parkin* genes. This allows us to say that this group observes the induction of mitophagy.

## 4. Discussion

In this work, we have determined the effect of alisporivir (non-immunosuppressive inhibitor of the MPT pore) on structural and functional changes in cardiac mitochondria in high-fat/streptozotocin-induced diabetes mellitus.

There are quite contradictory studies on the role of the MPT pore in the development of diabetes mellitus. Indeed, a number of studies have shown that MPT pore inhibitors reduce the risk of heart attack in diabetic animals [20,21]. However, other studies have found that MPT pore inhibitors do not have antidiabetic effects and fail to restore cardioprotection [22,39]. In our work, we also received rather contradictory data.

On the one hand, administration of alisporivir to diabetic animals (2.5 mg/kg/day) did not lead to a significant decrease in the level of glucose (fed state), triglycerides, and malondialdehyde in blood plasma, although we noted a tendency towards their recovery to control values. The same tendency to recovery was observed in the structural and functional characteristics of the heart—weight and heart rate. At the same time, one should note that during the glucose tolerance test, the administration of alisporivir showed an increase in the glucose clearance rate in diabetic animals. Previously, it was shown that knockout of cyclophilin D led to a similar effect in animals, which was associated with an increase in the rate of glucose uptake by skeletal muscle cells of mice [40].

At the same time, we noted that administration of alisporivir to diabetic animals partially prevented the DM-induced ultrastructural and functional alterations in heart mitochondria. Indeed, alisporivir normalized the structure of the heart mitochondria in mice. Administration of alisporivir to diabetic animals led to a decrease in vacuolization of organelles and restoration of the regular structure of the cristae. Moreover, we found that cardiac mitochondria of the DM + Ali group showed a significant increase in Ca^2+^ capacity in comparison with the mitochondria of diabetic animals. This suggests that alisporivir treatment of diabetic animals prevents MPT pore opening. In addition, alisporivir prevented the development of oxidative stress. In particular, the level of malondialdehyde in the mitochondria of DM + Ali mice significantly decreased in comparison with the level of the DM group and reached control values. Previously, other inhibitors of MPT pore opening have shown similar protective effects against the development of oxidative stress in the diabetic heart [20]. Possibly, these effects of alisporivir (prevention of MPT pore induction and the development of oxidative stress) underlie the normalization of the mitochondrial structure. On the other hand, suppression of the MPT pore assembly may promote restoration of Ca^2+^ homeostasis and cardiac activity. At the same time, the administration of alisporivir did not reveal a reliable restoration of the respiration process and oxidative phosphorylation of mitochondria. The respiration rate of cardiac mitochondria in state 3, as well as the respiratory control parameter of alisporivir-treated DM mice, did not significantly differ from these parameters in the DM group and were significantly lower than in the CTR group.

An important result of this work are changes in the level of mitochondrial DNA, as well as in the expression of genes responsible for mitochondrial biogenesis and mitophagy. According to the obtained and published data, the development of diabetes mellitus is associated with a reduction in the level of mitochondrial DNA. At the same time, the administration of alisporivir did not lead to the normalization of this parameter, and moreover, caused a further decrease in the level of mtDNA in the heart of diabetic animals. In addition, alisporivir reduced the amount of mtDNA in the heart cells of control animals. This suggests that alisporivir treatment reduces the number of mitochondria in heart cells. Additionally, if in the case of diabetes mellitus, a decrease in the number of damaged mitochondria may be a positive factor, then in healthy animals the similar effect of alisporivir is rather negative.

Reduction of mtDNA and, as a consequence, the number of mitochondria in a cell can occur as a result of suppression of biogenesis or induction of mitophagy. For example, it was previously shown that cyclosporin A, whose molecule only slightly differs from alisporivir, suppresses mitochondrial biogenesis in HepG2 cell culture [41]. In this work, we did not find any changes in the expression of the *Ppargc1a* gene encoding PGC-1α responsible for mitochondrial biogenesis. However, we observed a downward trend in the expression level of this gene in three experimental groups (CTR + Ali, DM, and DM + Ali) relative to the CTR group. One could speculate that such a decrease has an effect on a decrease in the number of mitochondria in the heart cells of these groups of animals. At the same time, the DM + Ali group showed a significant increase in the expression of *Pink1* and *Parkin* genes encoding the proteins responsible for the mitophagy. Thus, one could assume that the administration of alisporivir to diabetic animals leads to the activation of mitophagy and the removal of damaged mitochondria from the heart cells of diabetic animals.

## 5. Conclusions

The data obtained in this work allow us to draw the following conclusions. First, alisporivir at the concentration used (2.5 mg/kg/day) is not antidiabetic. At the same time, the tendency to restore a number of parameters (the level of glucose (fed state), malondialdehyde and plasma triglycerides, heart rate), as well as the fact that Ali-treated DM animals showed a significant increase in the rate of glucose clearance in the blood, allows us to hope that more high concentrations of this compound may have beneficial effects against the development of diabetic cardiomyopathy. Secondly, alisporivir partially restores the ultrastructure and functions of the heart mitochondria in diabetic animals. At the same time, the administration of alisporivir results in a decrease of the amount of mitochondrial DNA in both diabetic and control animals. In parallel with this, diabetic animals treated with alisporivir showed a significant increase in the expression of the *Pink1* and *Parkin* genes, encoding proteins responsible for mitophagy. Thus, alisporivir can reduce the number of damaged mitochondria in the diabetic heart. However, a decrease in the number of mitochondria in healthy animals may indicate negative effects of this compound. All this suggests that the use of this drug should be undertaken with care, bearing in mind multiple effects of alisporivir, a proper evaluation of which would require multivariate analysis.

## Figures and Tables

**Table 1:** List of gene-specific primers for RT-PCR analysis.

Gene	Forward (5′→3′)	Reverse (5′→3′)
*Nd4*	ATTATTATTACCCGATGAGGGAACC	ATTAAGATGAGGGCAATTAGCAGT
*Gapdh*	GTGAGGGAGATGCYCAGTGT	CTGGCATTGCTCTCAATGAC
*Ppargc1a*	CTGCCATTGTTAAGACCGAG	GTGTGAGGAGGGTCATCGTT
*Pink1*	TTGCCCCACACCCTAACATC	GCAGGGTACAGGGGTAGTTCT
*Parkin*	AGCCAGAGGTCCAGCAGTTA	GAGGGTTGCTTGTTTGCAGG
*Rplp2*	CGGCTCAACAAGGTCATCAGTGA	AGCAGAAACAGCCACAGCCCCAC

**Table 2:** Animal weights and biochemical characteristics of the blood in the experimental groups.

	CTR	CTR + Ali	DM	DM + Ali
Body weight, g	31.0 ± 0.8	29.7 ± 0.7	29.4 ± 0.7	29.2 ± 0.8
Heart weight, mg	152.9 ± 4.5	154.1 ± 4.1	128.3 ± 5.3 *	137.7 ± 4.0
BG (fed state), mM	10.4 ± 0.8	10.5 ± 1.1	20.5 ± 1.2 *	16.9 ± 1.3 *
Triglycerides, mM	1.61 ± 0.06	1.50 ± 0.07	1.96 ± 0.11 *	1.70 ± 0.11
[TBARS], nmol/mg	5.3 ± 0.8	5.4 ± 0.6	10.9 ± 1.4 *	9.4 ± 1.5 *

Values are given as mean ± SEM (*n* = 9). BG, blood glucose. * *p* < 0.05 compared to the control group (CTR).

## Data Availability

The data presented in this study are available on request from the corresponding author.
